# Hacking Trust: The Presence of Faces on Automated Teller Machines (ATMs) Affects Trustworthiness

**DOI:** 10.3390/bs11060091

**Published:** 2021-06-21

**Authors:** Giulio Gabrieli, Sarah Ng, Gianluca Esposito

**Affiliations:** 1 Psychology Program, Nanyang Technological University, Singapore 639818, Singapore; giulio001@e.ntu.edu.sg (G.G.); SNG073@e.ntu.edu.sg (S.N.); 2 Lee Kong Chian School of Medicine, Nanyang Technological University, Singapore 308232, Singapore; 3 Department of Psychology and Cognitive Science, University of Trento, 38068 Trento, Italy

**Keywords:** Halo Effect, trustworthiness, face perception

## Abstract

Trustworthiness is a core concept that drives individuals’ interaction with others, as well with objects and digital interfaces. The perceived trustworthiness of strangers from the evaluation of their faces has been widely studies in social psychology; however, little is known about the possibility of transferring trustworthiness from human faces to other individuals, objects or interfaces. In this study, we explore how the perceived trustworthiness of automated teller machines (ATMs) is influenced by the presence of faces on the machines, and how the trustworthiness of the faces themselves is transferred to the machine. In our study, participants (N = 57) rated the trustworthiness of ATMs on which faces of different age, gender, and ethnicity are placed. Subsequently, the trustworthiness of the ATMs is compared to the trustworthiness ratings of faces presented on their own. Results of our works support the idea that faces’ trustworthiness can be transferred to objects on which faces are presented. Moreover, the trustworthiness of ATMs seems to be influenced by the age of presented faces, with ATMs on which children faces are presented are trusted more than the same machines when adults’ or elders’ faces are presented, but not by the ethnicity (Asian or Caucasian) or gender (male or female) of presented faces.

## 1. Introduction

The Halo Effect is a cognitive bias in impression formation by which the general evaluation of a strangers’ attributes is based on the evaluation of a single attribute [1]. The aesthetic appearance is generally used in interaction with strangers as the basis for the estimation of other traits, that are not related to the aesthetics. For example, good-looking individuals may be perceived as intelligent, smart, kind, and competent, despite the fact that these traits are unrelated to physical attractiveness. Known to be intuitive, constant and pervasive [2,3,4,5,6], the Halo Effect has been widely investigated by researchers in the fields of Computer Science, Empirical Aesthetics and Social Psychology [7,8,9,10].

The relations between individuals’ aesthetic appearance and perceived trustworthiness (aesthetics × trustworthiness) has been especially studied since the beginning of the twentieth century [7]. Trustworthiness—an “umbrella” trait that is fundamental to social perception [11]— has diverse implications in numerous life domains. For example, when assessing another person’s ill or good intentions, we rely on the perceived trustworthiness to decide how to proceed in an interaction.

Several works demonstrate the existence of a relation between aesthetic appearance and perceived trustworthiness [12,13,14], and the strength of the Halo Effect between these two construct was further confirmed in a review conducted by Eagly et al. [15]. However, the study of individuals’ aesthetic appearance is not important exclusively for social interactions. The exploitability of individuals’ appearance gained special focus in marketing and advertisement research [16], with a large body of work that investigated the effectiveness of marketing campaigns and advertisement with regards to the presence of different individuals. Gilly [17], for example, investigated the gender role in Television advertisement in the U.S., Australia, and Mexico, revealing differences in the way and frequency in which male and female were depicted, especially with reference to the advertised product. Highlights of such works include the fact that women were more likely to appear in the advertisement of products meant for women or for both the gender, but not for men’s products. Similarly, the employment of actors of different ethnic background has been studied [18,19], especially for what concerns the role of minorities. Over the years, two frameworks have been proposed to investigate the impact of race on consumers’ evaluations of advertising: the ingroup bias theory—whereby members of the ingroup will be evaluated more favourably than members of the outgroup—and the polarized appraisal theory—for which members of the outgroup will be evaluated more extremely, both positively or negatively, than members of the ingroup—. An experimental study conducted by Qualls and Moore [20] revealed that the ingroup bias theory is better than the polarized appraisal theory at explaining the effect of ethnicity in consumers’ evaluation of advertising. However, the depiction of specific ethnic groups in advertising and commercial have been proven to evolve over time. A longitudinal study by North and Millard [21] on children appearance in South African magazines’ advertisements before and after the apartheid revealed an increase in recent years of the portrayal of children of different races, reflecting the sociopolitical changes that took place in the country.

The aesthetic appearance of children is of particular interest in psychology, and especially in social psychology. Children have in fact a set of specific physical traits, such as big head, big round eyes, small faces and short limbs that evoke protective caregiving behaviors in adults [22,23]. Defined as Baby Schema, this set of traits has been widely investigated to understand adults’ responses and behavior when children and children faces are presented [24,25], and has been proven to be consistent across cultures and ethnicities [14,23].

Not only the portrayal of individuals of different races changed in frequency and context over time, but also the strength of the Halo Effect between aesthetic appearance and perceived trustworthiness have been demonstrated to be unstable over time. A recent work [14] investigated the stability over time of the Halo Effect, by presenting participants with pictures of faces of different age, gender, and ethnicity, and asking how much they (i) found the face aesthetically pleasant and (ii) how much they trusted the person. Results revealed that the strength of the relation between aesthetics and perceived trustworthiness of strangers faces changed during the COVID-19 pandemic outbreak. The importance to focus on faces is crucial for their primary role in social interaction [23,26], however, most of the work on trustworthiness and human appearance focused on full bodies, and not specifically on faces [16]. With the increasing use of social media and digital platform, the focus on faces is of extreme interest, especially considering the wide adoption of human faces as profile pictures [27], as well as to promote brands and services [16]. Unfortunately, little is known about the possibility of transferring the Halo Effect from human faces to other objects [28,29,30]. In a study from Guthrie et al. [30], for example, it has been found that the aesthetic appearance of faces influence the perceived competence of a brand, while Fleck et al. [31] reported that the presence of a spokesperson can help giving human values to a brand. However, nothing is now about the quantitative impact of the presence of a human face on the perceived trustworthiness of an object or a brand.

In this work, we aim at investigating how the presence of human faces affects the perception of trustworthiness of automated teller machines (ATMs). Given the previously reported importance of the ethnicity [32] and gender [12,13,14] of presented faces on perceived trustworthiness, as well as the specificity of children faces [14], faces of different gender, ethnic groups (Caucasian and Asians) and age (children and adult) will be studied. Moreover, to assess the stability of the Halo Effect over time, the strength of the Halo will be measures for each participants in two different sessions.

In line with recent works on the Halo Effect [14], we hypothesize that (H1) the perception of the trustworthiness of an ATM does not significantly differ after a time delay (7 days). Moreover, (H2) in line with the results of Qualls and Moore [20], we expect a stronger higher perceived trustworthiness for ingroup individuals, of similar age and gender. Finally, (H3) we predict a higher perceived trustworthiness on ATMs when children faces are displayed vs. when adult or elderly faced are used for this purpose. These last two hypotheses—H2 and H3— are at least partially self exclusive, as a preference for ingroup individuals, of similar age and gender would support the ingroup bias theory as the preferred pathway for which the Halo Effect occurs in adults, while results in support of a preference for children faces, which would back up the Baby Schema as the preferred model driving the Halo Effect of trustworthiness.

For what concerns the transposition of the Halo Effect from human faces to objects on which faces are presented, data collected for this work (ratings of objects) are compared to data collected in two previous studies that employed the same set of faces [14]. We hypothesize (H4) a strong correlation between the Halo Effect of faces (measured as the Pearsons’ correlation between perceived aesthetics and perceived trustworthiness ratings of human faces) and the Halo Effect of the ATMs (measured as the Pearsons’ correlation between perceived aesthetics and perceived trustworthiness ratings of the ATMs).

## 2. Materials and Methods

### 2.1. Study Design

#### 2.1.1. Stimuli

**Figure 1:**
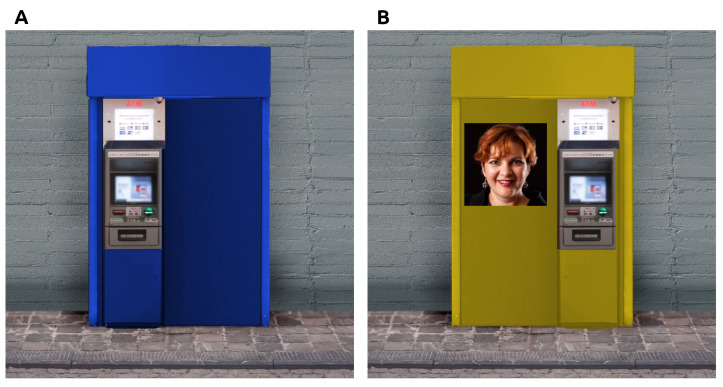
Examples of images employed in this study. (**A**): example of ATM with interface on the left side, with no faces added. (**B**): example of ATM with interface on the right side, with a face placed on the left side.

Multiple versions of an ATM were generated using GIMP, an open source image manipulation software. From the photography of an ATM, two versions were first generated, one with the screen and keypad on the left side, and one with the screen and keypad on the right side. The empty space on the opposite side of the interface was enlarged, in order to fit a squared image of 256 × 256 pixels. Then from the two versions (left and right), four more version in different colors—green, red, yellow, and blue—. Subsequently, front facing images of faces were placed on the images of the ATMs. A total of 96 images were generated, using faces of different ages (32 children’s faces, 32 adults’ faces, 32 elders’ faces), genders (48 males, 48 females) and ethnicities (48 Asians, 48 Caucasians), such that for each combination of age, gender, and ethnicity, a total of 8 faces are presented. Faces’ images were selected from the FFHQ Dataset [33], a dataset containing 70,000 high-quality (1024 × 1024) images published on Flickr under different creative commons and public domain licenses (Creative Commons BY 2.0, Creative Commons BY-NC 2.0, Public Domain Mark 1.0, Public Domain CC0 1.0, or U.S. Government Works license) and used in previous works [33,34,35,36], including works on the Halo Effects for which ratings of aesthetics and trustworthiness of the faces were collected [14]. Examples of generated images are reported in Figure 1.

Several works demonstrate the existence of a relation between aesthetic appearance and perceived trustworthiness [12,13,14], and the strength of the effect was further confirmed in a review [15].

#### 2.1.2. Procedure

The method was adapted from a previous work on the Halo Effect [14]. Manipulated images containing faces, as well as the eight (N = 8) depicting ATMs of different colors with the HID (Human Interface Design) on either the left or right side were presented in random order via an online survey, together with three attention checks. Each image was presented along with two questions, measuring the perceived aesthetic appearance and the perceived trustworthiness of the ATMs on a 100-points Likert scale, anchored from 1 being “not at all” to 100 = “extremely”. Stimuli and questions were presented in a random order, with no time constraints. Participants were asked to complete the survey twice, with an interval of seven days within the two sessions.

#### 2.1.3. Aesthetics and Trustworthiness Ratings of Faces

Aesthetics and trustworthiness ratings of faces are drawn from two previous studies that employed the same set of faces [14] and in which participants were asked to rate the perceived aesthetics and perceived trustworthiness of each face. The first set of ratings (“First Face Dataset”) has been collected from 380 participants (N = 145 Asians, N = 235 Caucasians) between August 2019 and April 2020, while the second set of ratings (“Second Face Dataset”) has been collected from 289 participants (N = 144 Asians, N = 145 Caucasians) between September and December 2020. The two datasets are here employed to verify the extent to which the perceived aesthetics and trustworthiness of faces is transferred to the machine itself, when faces are presented on the machine. The two dataset are available online on the Data Repository of the Nanyang Technological University [37,38].

### 2.2. Analytic Plan

The analytic plan for the current study was pre-registered on the Open Science Framework [39], after ethical approval was granted. To test the three hypotheses, a mixed between-within subject ANOVA is used. The required number of participants was estimate trough a power analysis, conducted in G*Power [40,41], that for the type of test, and for the selected values for alpha and power (α = 0.05, power = 0.95) revealed that sixty (N = 60) participants are required. To take into account possible drop off, errors, and participants excluded due to failures in passing attention checks, more than 60 participants were targeted.

For what concerns the hypotheses testing, a single mixed between-subject ANOVA is employed to test the three hypotheses. For our first hypothesis to be verified, which is that the perception of the trustworthiness would not differ significantly across the two sections, no significant effect of the presentation time (1st vs 2nd session) is expected. For what concerns the subsequent two hypotheses (H2) and (H3), significant main effects are expected for what concerns the age of presented faces for both H2 and H3, while additional main effects of Ethnicity and Gender of presented faces, as well as an interaction effects of age, gender, and ethnicity of presented faces. Finally, for H4 to be verified, which is the transposition of the Halo Effect from faces to objects, a significant correlation between the Halo Effect of faces and the Halo Effect of ATMs is expected.

### 2.3. Participants

The protocol of the study was approved by the Internal Review Board of the Nanyang Technological University (IRB-PSY-2020-016) and conducted according to the declaration of Helsinki. Informed consent was obtained from all the participants before the study. Participants (N = 86) voluntarily participated and were recruited through the Nanyang Technological University’s School of Social Sciences Research Participation System or via different social media. All the participants were residing in Singapore at the time of participation and identified themselves as of Asian ethnicity. Only data from participants who correctly replied to all the attention checks were kept. The final dataset consists therefore of the data of fifty-seven (N = 57, mean age = 22.62±1.95, 26 females) participants [42].

## 3. Results

**Figure 2:**
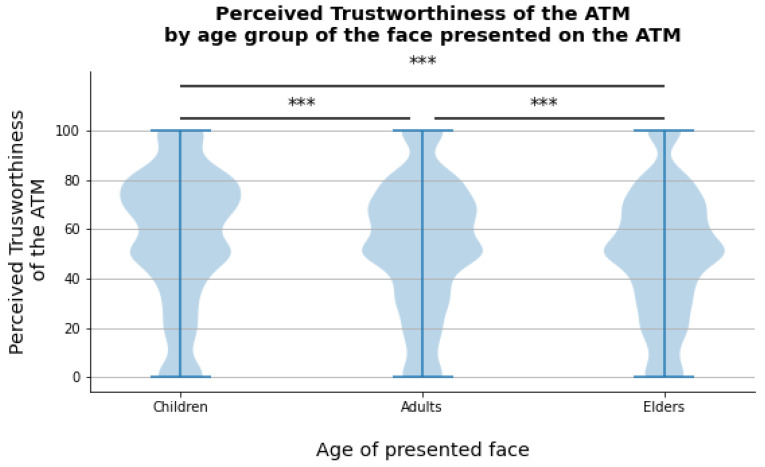
Distribution of Perceived Trustworthiness scores by age group (child, adult, or elder) of faces presented on ATMs. *** *p* < 0.001.

To exclude any possible cofound of the color of the ATM and of the position of the picture within the ATM (left or right side), a Type II ANOVA has been conducted in R (Version 3.6.3, GNU/Linux 64 bit, package “car”). Results revealed no main nor significant effects of color and position on ATMs’ perceived trustworthiness. To test the first three hypotheses (H1, H2, H3), a Type II ANOVA has been employed. Results, reported in Table 1, reveals no main effect nor interaction effect of Time (*F*-value = 1.6315, *p*-value = 0.20153), suggesting a stability in trustworthiness judgments over the two sessions, therefore supporting our first hypothesis. Moreover, results reveal a main effect of the Age of presented faces (*F*-value = 66.582, *p*-value = 2 × 10^−16^. $\eta_{p}^{2}=0.01$), but not of the gender of the face (recoded as Same or Different from the participants’ gender, *F*-value = 0.0025, *p*-value = 0.96042) or of the Ethnicity (recoded as Same or Different from the participants’ ethnicity, *F*-value = 3.7301, *p*-value = 0.05347), not supporting the ingroup bias theory, as well as our second hypothesis (H2). A post-hoc analysis on the differences in perceived trustworthiness by the age group of presented faces highlight the existence of significant differences between the perceived trustworthiness of child’s and adults’ faces (*t*-value = 5.81, corrected *p*-value = 1.95 × 10${}^{-8}$, Cohen’s d = 0.13), as well as between child’s and elders’ faces (*t*-value = 11.49, corrected *p*-value = 7.63 × 10${}^{-30}$, Cohen’s d = 0.26) and adults’ and elders’ faces (*t*-value = 5.76, corrected *p*-value = 2.63 × 10${}^{-8}$, Cohen’s d = 0.13), with children faces rated on the average significantly more trustworthy (mean trustworthiness 57.10= ±27.28) than adults’ (mean trustworthiness = 53.50 ± 26.11) and elders’ faces (mean trustworthiness = 50.05 ± 25.59), as shown in Figure 2. In light of these results, our third hypothesis, which is that ATMs’ perceived trustworthiness is higher when children faces are presented, is confirmed.

**Figure 3:**
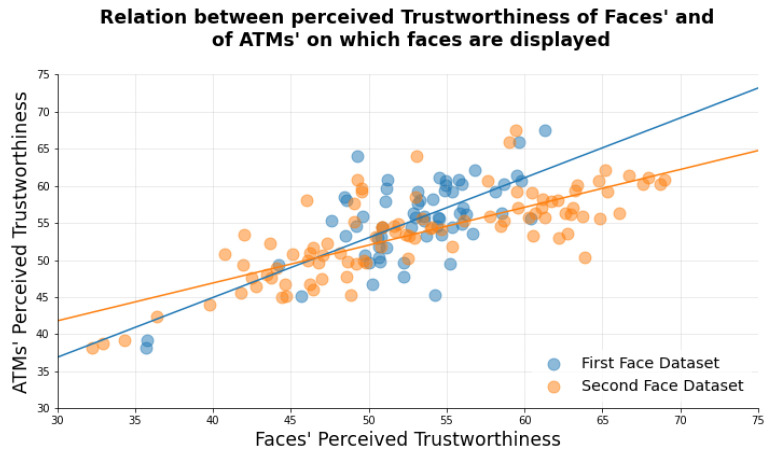
Relation between perceived Trustworthiness of Faces’ and of ATMs’ on which faces are displayed by faces’ ratings dataset (First Face Dataset in blue, Second Face Dataset in Orange).

For what concerns the possible transferability of the Halo Effect from faces to ATMs, we first verified the existence of significant differences between the trustworthiness ratings of ATMs when no faces are presented, as compared to when faces are presented on the machines. A two-tailed paired *t*-test revealed significant differences between the ratings (*t*-value = 77.012, *p*-value < 0.001). Subsequently, we verified the relation between the perceived trustworthiness of faces when presented alone and the perceived trustworthiness of ATMs when the same faces are presented on them (Figure 3). Significant positive correlations (First Face Dataset: Pearson’s r = 0.69, *p*-value = 3.84 × 10${}^{-10}$; Second Face Dataset: Pearson’s r = 0.78, *p*-value = 1.37 × 10${}^{-20}$) were found between the ratings of the faces collected in previous works and the trustworthiness ratings of ATMs collected in the current study, and the two correlation did not significantly differ from each other (z-value = −1.198, *p*-value = 0.115).

## 4. Discussion

In this work, we investigated how the presence of human faces affects the perception of trustworthiness of ATMs. To the best of our knowledge, this is among the first studies that consider the possibility that the Halo Effect is transferable from human faces to other objects and interfaces on which they are displayed. The effect of time on trustworthiness ratings, as well as of the possible differences due to the age, gender, and ethnicity of presented faces have been inspected using an ANOVA. Results revealed no effect of time on trustworthiness ratings, suggesting an overall stability of the ratings in measures collected 7 days apart from each other. Moreover, we found a significant main effect of the age of presented faces on perceived trustworthiness ratings, but not of the gender or ethnicity, at least for what concerns the genders and ethnic groups considered in this study (male/female, Asian/Caucasian). These results support our first hypothesis (H1), which is that trustworthiness ratings ATMs do not significantly differ after a time delay (7 days), but do not support our second hypothesis (H2), which is that rater should have higher trust toward individuals of their ingroup, of their same gender and of similar age. However, results support our third hypothesis (H3), which is that rater should have higher trusts for ATMs on which faces of children are displayed. In fact, we found that ATMs on which children faces are displayed received significantly higher trustworthiness ratings, as compared to machines on which adults’ or elders’ faces were placed. Taken together, these findings suggest that when no external manipulation (e.g., priming to alter ATMs or faces’ trustworthiness) is employed, trustworthiness ratings are stable over time. Moreover, our findings suggest that when it comes to the Halo Effect, the Baby Schema—preference for children faces— has a more prominent role in shaping trustworthiness judgments, as compared to the ingroup bias theory—preference for individuals of similar age, gender, and ethnicity—. The evolutionary perspective can suggest a possible explanation for these results. Adult individuals may be more prone to have more confidence in children as compared to adults, given the primary role children have in the survival of the species [14].

For what concerns the possible transition of faces’ perceived trustworthiness ratings to ATMs perceived trustworthiness, which is our fourth hypothesis (H4), our results demonstrate a strong correlation between the trustworthiness ratings of faces and the trustworthiness ratings of ATMs. The comparison of the perceived trustworthiness ratings of faces, obtained in previous works, and the perceived trustworthiness of ATMs on which the same faces have been photo manipulated. We found strong significant correlation between the trustworthiness ratings of faces and the trustworthiness ratings of the ATMs. While data from the faces’ studies were collected in different moment in time, we found no significant differences between the strength of the correlation of ATMs’ and faces’ trustworthiness of the two studies. Taken together, these findings support the idea that the Trustworthiness judgments can be transferred from one element to another, in this case from faces to ATMs. This possibility opens to several implication. Designers, for example, could take advantage of the transferability effect by placing pictures of good looking and trustworthy individuals in advertisements to increase the trust toward a product, or in web application to increase the perceived trustworthiness of a web service. Similarly, given the specificity of children’s faces, younger kids could be exploited to increase adults’ perceived trustworthiness. For example politicians may pose next to infants and children in an attempt to increase their trustworthiness as seen as by possible electors. Moreover, results of this work open a possible field of study for researchers investigating the Halo Effect, and more specifically on how the Halo Effect of a face can be transferred to different objects and interfaces. Future works should consider studying the transferability of trustworthiness judgments in different settings, on both physical and digital devices, as well as across individuals.

Despite our best efforts to limit any possible confound in our results, this study is affected by some limitations. First, the pool of participants selected for this study is limited to Asian young-adults, within a narrow age-range. While from our previous works, as well as from other published works, we don’t expect differences between Asians and individuals of other ethnicities, future studies should replicate the study with a more variegated sample in term of Age and Ethnicity. Additionally, the number of stimuli employed here is limited to faces that have been previously tested on their own, and the number of different combination of color of the ATMs on which faces are presented is limited. Future studies should employed different images of both faces and ATMs (e.g., ATMs on different backgrounds). Moreover, while this study employed still images of human faces, interactive interfaces can make use of dynamic and animated animations or videos. Future works should consider the impact of such dynamic mediums not only on ATMs, but also on other automated devices that communicate to customers using an interactive interface. Finally, in our experiment we assumed that ATMs have available space on them which can be used for placing advertisements. However, in real-life scenarios, ATMs are available in different shapes and sizes, therefore the ideal setup described in this study may not be replicable on all the possible range of devices. Additionally, the location, position, and presence of others around the machine may influence users’ perceived trustworthiness of the device. Future works should consider how external events may influence ATMs trustworthiness, both in the presence and absence of others’ faces.

## 5. Conclusions

In this study, we investigated how time, as well as age, ethnicity and gender of faces presented on automated teller machine affects the perception of trustworthiness of the automated teller machine themselves. Results of our work suggest that the perception of trustworthiness of ATMs is stable over time, but influenced by the presence of images of faces on the machines. Individuals appear to be influenced by the Age of presented faces, but not by the ethnicity (Asian or Caucasian) or gender (male or female) of presented faces. More specifically, we found that the perceived trustworthiness of an ATM machine is higher when faces of children are presented on the machine itself. Our results could help designer increase the trustworthiness of their products, and especially of critical aspects of services, as well as open a discussion on the ethical practices of employing children in the advertisements of products, or of persons, such as in the case of public figures posing with infants and children.

## Figures and Tables

**Table 1:** ANOVA Table.

	*F*-Value	*p*-Value
Age	66.5820	2 × 10^−16^ ***
Gender	0.0025	0.96042
Ethnicity	3.7301	0.05347
Time	1.6315	0.20153
Age×Gender	0.4683	0.62608
Age×Ethnicity	0.7429	0.47578
Gender×Ethnicity	0.0636	0.80094
Age×Time	1.2860	0.27641
Gender×Time	0.0187	0.89131
Ethnicity×Time	0.3463	0.55622
Age×Gender×Ethnicity	0.2890	0.74906
Age×Gender×Time	0.0600	0.94180
Age×Ethnicity×Time	0.0085	0.99153
Gender×Ethnicity×Time	0.0227	0.88014
Age×Gender×Ethnicity×Time	0.0839	0.91949

*** *p*-value < 0.001.

## Data Availability

The dataset generated for this study is available online on the Data Repository of the Nanyang Technological University [42].

## Abbreviations

The following abbreviations are used in this manuscript:

ATM	Automated Teller Machine
HID	Human Interface Device
GIMP	GNU Image Manipulation Program
FFHQ	Flicker Faces High Quality
